# Essential Thrombocythemia in a Nonagenarian Presenting With Acute Myocardial Infarction

**DOI:** 10.7759/cureus.9955

**Published:** 2020-08-23

**Authors:** Junya Tanabe, Madoka Yamaguchi, Hirotomo Sato, Akihiro Endo, Kazuaki Tanabe

**Affiliations:** 1 Cardiology, Shimane University Faculty of Medicine, Izumo, JPN

**Keywords:** myocardial infarction, nonagenarian patient, essential thrombocythemia

## Abstract

Essential thrombocythemia (ET) is a chronic Philadelphia-negative myeloproliferative neoplasm (MPN) characterized by clonal thrombocytosis and an increased risk of arterial and venous thrombosis. Because ET has a low probability of progressing to acute leukemia or myelofibrosis and its prognosis is determined by thromboembolic or bleeding symptoms, the treatment of this disease is aimed at preventing vascular events. We encountered a nonagenarian patient with ET who presented with acute myocardial infarction with ST-segment elevation. In the present case, the patient was 91 years old, and antithrombotic agents were required after the placement of drug-eluting stent. Therefore, we decided not to perform cytoreductive therapy because the risk of bleeding is higher. Very elderly patients with ET are at an increased risk of thrombotic and hemorrhagic events. The risk-benefit of antithrombotic therapy should be considered individually.

## Introduction

Thrombocytosis (platelet count ≥ 450,000/μL) is frequently encountered in clinical practice, with a prevalence of 1.9% in large studies involving outpatients aged 65 years and older. In a retrospective study of patients with thrombocytosis, primary thrombocytosis (myeloproliferative neoplasm [MPN]) accounted for 5.2% of the cases, and other causes included infection (47.9%), trauma or postoperative conditions (24.5%), other cancers (10.7%), and iron deficiency anemia (7.4%) [1]. Essential thrombocythemia (ET) is a chronic Philadelphia-negative MPN characterized by clonal thrombocytosis and an increased risk of arterial and venous thrombosis [2]. Because ET has a low probability of progressing to acute leukemia or myelofibrosis and its prognosis is determined by thromboembolic or bleeding symptoms, the treatment of this disease is aimed at preventing vascular events [3]. Herein, we report a case of ST-elevation myocardial infarction (STEMI) in a nonagenarian patient with ET.

## Case presentation

**Figure 1:**
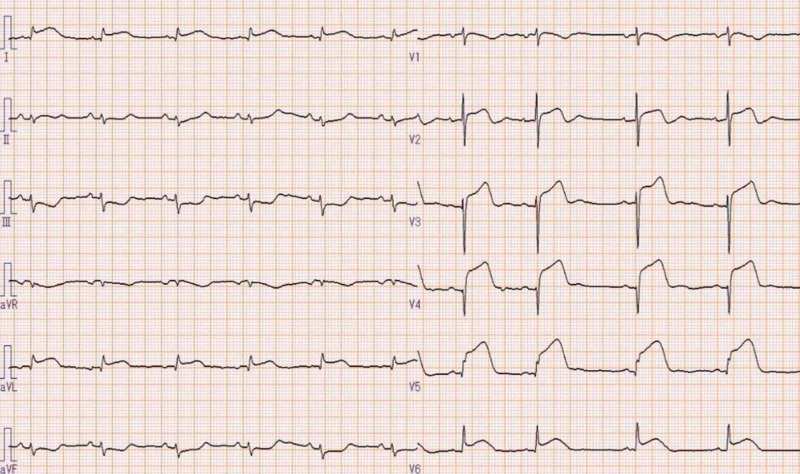
Twelve-lead electrocardiogram shows ST-segment elevation in I, aVL, and V2-V6 with ST-segment depressions in leads III and aVF.

The patient was a 91-year-old woman who was transferred to our hospital complaining of chest and back pain. A 12-lead electrocardiography (ECG) showed ST-segment elevation in I, aVL, and V2-V6 with ST-segment depressions in leads III and aVF (Figure 1).

**Figure 2:**
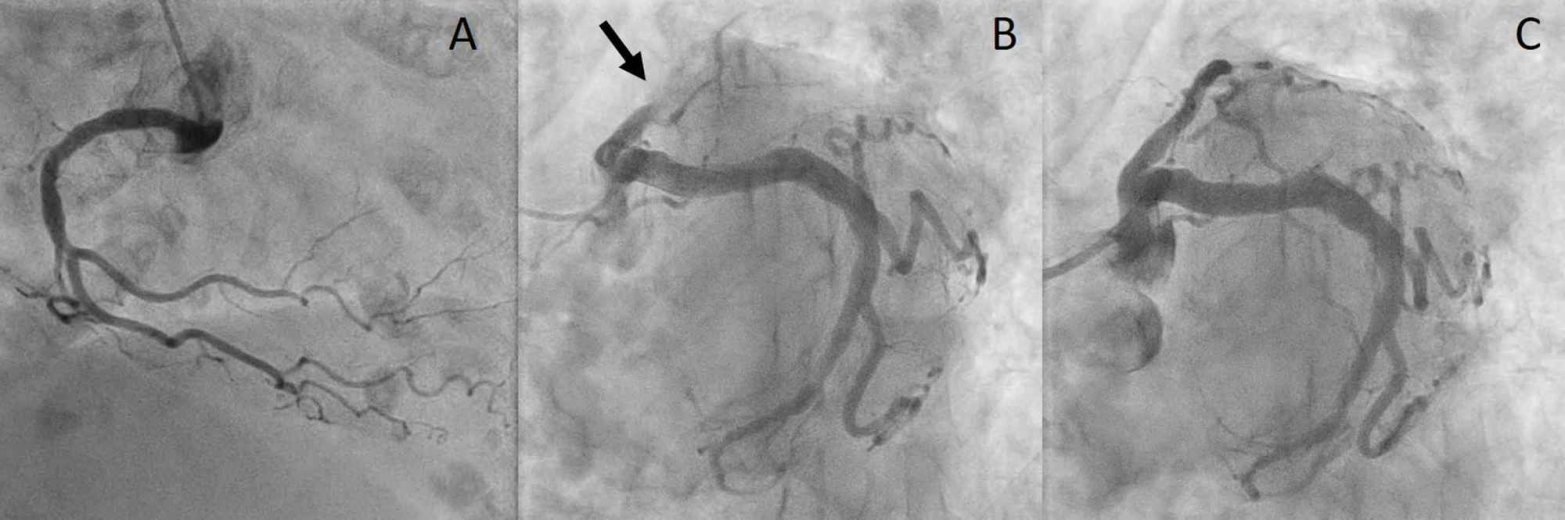
Emergent coronary angiography reveals no significant right coronary artery stenosis (A) and complete occlusion of the proximal left anterior descending artery with normal left circumflex artery without significant lesions (B, arrow). Percutaneous coronary intervention (thrombus aspiration and placement of a drug-eluting stent) was successful (C).

The patient had a medical history of type 2 diabetes mellitus, hypertension, and dementia, and an increase in platelet count was indicated four months before the admission. Physical examination on admission revealed the following: blood pressure, 120/71 mmHg; heart rate, 63 beats/min; and oxygen saturation, 93% with 2 L of oxygen. No heart murmur was heard, and respiratory sounds were clear. Laboratory investigation revealed a marked increase in the platelet count to 574,000/μL. The level of troponin I was elevated to 0.48 ng/mL (<0.04 ng/mL). Transthoracic echocardiography revealed a left ventricular ejection fraction of 40%, and apical akinesia. Emergent coronary angiography revealed complete occlusion of the proximal left anterior descending artery; therefore, an emergent percutaneous coronary intervention (thrombus aspiration and placement of a drug-eluting stent) was performed (Figure 2).

The procedure was successful without major complications. Aspirin 100 mg and clopidogrel 75 mg per day were chosen as the antithrombotic agents. Analysis of JAK2 revealed a heterozygous mutation, Val-617-Phe, and the patient was diagnosed with ET. Because the patient was very old and her platelet count was stable, cytoreductive therapy was not administered in view of bleeding tendency. Cardiac rehabilitation progressed smoothly, and the patient was discharged from the hospital without any complications.

## Discussion

ET is a disease in which neoplastic proliferation of pluripotent stem cells causes hyperplasia of megakaryocytes in the bone marrow, leading to thrombocytosis [1]. The estimated rate of occurrence of ET is 1.2-3.0 per 100,000 people per year [4,5]. The probability of transformation of ET to acute leukemia or myelofibrosis is 2.9%, and the prognosis is determined by thromboembolism and bleeding symptoms [6]. The presence of JAK2 V617F mutation is associated with a significantly increased risk of thrombosis [1]. Bleeding in patients with ET is in some cases associated with acquired von Willebrand’s disease, which results from a selective loss of large von Willebrand’s factor multimers. Therefore, the goals of ET treatment are to prevent thrombotic and hemorrhagic complications and to relieve symptoms. Patients with risk factors for cardiovascular disease, such as smoking, diabetes, hypertension, and hyperlipidemia, should be actively treated for these complications. Cytoreductive therapy, such as with hydroxyurea, is recommended for patients at high risk. For high-risk patients, a platelet count under 400,000/μL has been recommended. Limited information is available regarding treatment practice in very elderly patients with ET. A study on the efficacy and safety of cytoreductive therapy in patients older than 80 years with ET showed that it was well tolerated and effective [7]. In this case, the patient was 91 years old, which is a very advanced age, and antithrombotic agents were required after the placement of drug-eluting stent. Therefore, we decided not to perform cytoreductive therapy because the risk of bleeding is higher, and there is a lack of clear evidence for its efficacy in very elderly individuals; another reason was that the platelet count was stable at 500,000/μL.

## Conclusions

We encountered a nonagenarian patient with ET who presented with STEMI. Very elderly individuals are at an increased risk of thrombotic and hemorrhagic events. There are few data for the very elderly patients with ET; therefore, the risk-benefit of antithrombotic therapy should be considered individually, and the treatment plan should be carefully formulated.
